# Chiral topographic instability in shrinking spheres

**DOI:** 10.1038/s43588-022-00332-y

**Published:** 2022-10-24

**Authors:** Fan Xu, Yangchao Huang, Shichen Zhao, Xi-Qiao Feng

**Affiliations:** 1 Institute of Mechanics and Computational Engineering, Department of Aeronautics and Astronautics, Fudan University, Shanghai, P. R. China; 2 Institute of Biomechanics and Medical Engineering, AML, Department of Engineering Mechanics, Tsinghua University, Beijing, P. R. China

**Keywords:** Mechanical engineering, Soft materials, Theory and computation, Biological physics, Applied mathematics

## Abstract

Many biological structures exhibit intriguing morphological patterns adapted to environmental cues, which contribute to their important biological functions and also inspire material designs. Here, we report a chiral wrinkling topography in shrinking core–shell spheres, as observed in excessively dehydrated passion fruit and experimentally demonstrated in silicon core–shells under air extraction. Upon shrinkage deformation, the surface initially buckles into a buckyball pattern (periodic hexagons and pentagons) and then transforms into a chiral mode. The neighbouring chiral cellular patterns can further interact with each other, resulting in secondary symmetry breaking and the formation of two types of topological network. We develop a core–shell model and derive a universal scaling law to understand the underlying morphoelastic mechanism and to effectively describe and predict such chiral symmetry breaking far beyond the critical instability threshold. Moreover, we show experimentally that the chiral characteristic adapted to local perturbation can be harnessed to effectively and stably grasp small-sized objects of various shapes and made of different stiff and soft materials. Our results not only reveal chiral instability topographies, providing fundamental insights into the surface morphogenesis of the deformed core–shell spheres that are ubiquitous in the real world, but also demonstrate potential applications of adaptive grasping based on delicate chiral localization.

## Abstract (web summary)

This study reports a chiral instability topography in highly deformed core–shell spheres. A core–shell model and a scaling law are developed to understand its morphoelastic mechanism, which helps the design of a nature-inspired smart topographic gripper based on chiral localization.

## Main

Morphological pattern formation across length scales is energetically favourable for thin-walled living matter such as fruits^1,2^, vegetables^3^, leaves^4–6^, embryos^7^, organs^8^, tumours^9^ and brains^10^, where spontaneous symmetry breaking during growth or dehydration is normally considered to be a crucial factor in their complex wrinkling topography^6,11,12^. For example, pollen grains of angiosperm flowers exhibit self-folding when exposed to a dry environment to prevent further desiccation^13^. Growth-induced residual stress accumulates during tumour progression, driving the global buckling collapse of blood and lymphatic vessels, which makes the vascular delivery of anticancer drugs ineffective^9^. Symmetry breaking in evolving wrinkling patterns during brain development results in the thickness difference between gyri and sulci, which is closely linked to neurodevelopment disorders such as lissencephaly, polymicrogyria, autism spectrum disorders and schizophrenia^14^. In terms of its practical use, symmetry breaking in the formation of surface morphology patterns has found ever-increasing applications in various fields, such as micro/nanofabrication of flexible electronic devices^15,16^, surface self-cleaning and anti-fouling^17^, synthetic camouflaging skins^18^, shape-morphing soft actuators^19^ and adaptive aerodynamic drag control^20^. The precise prediction, control and manipulation of reversible instability morphologies would be key for relevant applications.

**Fig. 1:**
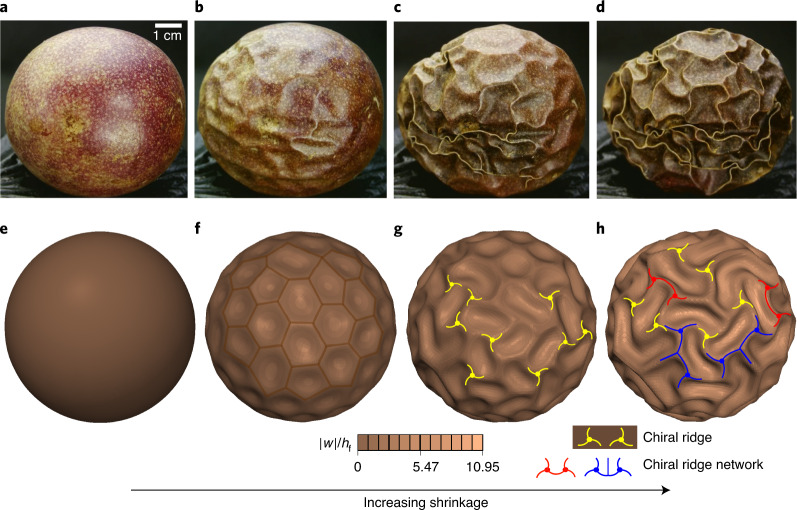
Evolution of wrinkling topography in excessive dehydration of deformed passion fruit. **a**–**h**, Natural observations (**a**–**d**) and model predictions (**e**–**h**) on day 1 (**a**,**e**), day 2 (**b**,**f**), day 4 (**c**,**g**) and day 7 (**d**,**h**). Upon shrinkage, the core–shell spheres first buckle into a buckyball pattern (periodic hexagons and pentagons in **b** and **f**) and then transform to a chiral ridge (**g**) and eventually to a ridge network (**h**) with the coalescence of neighbouring chiral ridges. The core experiences isotropic shrinking (Supplementary Sections I and II and Video 1).

Prior works^3,12,21–23^ on morphological pattern formation in stressed spherical core–shells, a typical structure omnipresent in nature and industrial technologies, have demonstrated a variety of intriguing topographies such as dimple, buckyball and labyrinth modes. Here, we report a chiral instability topography in core–shell spheres. We observed that a drying passion fruit (*Passiflora edulia* Sims) initially buckles into a periodic buckyball pattern consisting of hexagons and pentagons, evolving into a chiral mode, and forms intriguing chiral topological networks upon excessive shrinkage (Fig. 1). Inspired by this natural phenomenon, we explored, both theoretically and experimentally, the morphological pattern formation and evolution of highly deformed core–shell spheres, especially the emergence of a chiral pattern and chiral ridge networks with symmetry breaking at the advanced bifurcation. We established a mathematical model and a scaling law to capture the chiral instability of core–shell spheres and explored a potential application of perturbation-adaptive chiral localization.

## Results

### Theory

**Extended Data Fig. 1:**
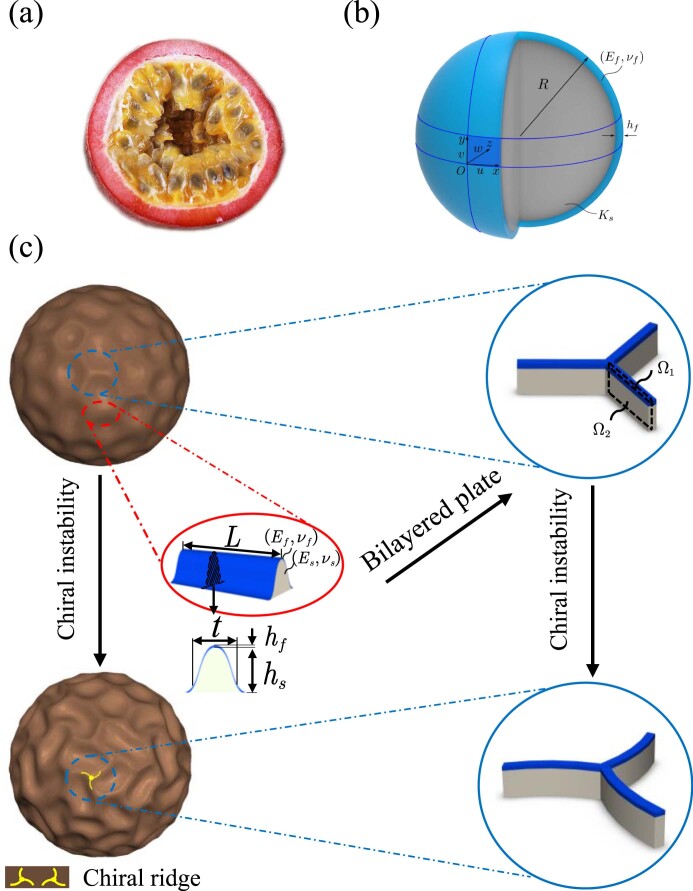
Passion fruit (Passiflora edulia Sims). (a) Cross section. (b) Geometry of a core-shell sphere. (c) Schematic of chiral buckling of a Y-shaped cellular representative layered plate.

To understand the underlying mechanism and to effectively predict the morphogenesis process, we consider an elastic spherical shell supported by a soft core. Upon shrinkage, the shell buckles elastically to relieve the compressive stress while the core concurrently deforms to maintain perfect bonding at the interface. In shallow shell theory^24^, the coordinates of the core–shell system can be Cartesian in a tangent plane (or curvilinear and orthogonal). This framework can only describe a part of the spherical geometry (Extended Data Fig. 1), but it is competent here for theoretical analyses. The thickness of the surface layer is denoted by *h*_f_, while the radius of the system is represented by *R*. The Young’s modulus and Poisson’s ratio of the surface layer are denoted by *E*_f_ and *ν*_f_, respectively, while *E*_s_ and *ν*_s_ are the corresponding material properties of the soft core. The elastic strain energy *Π*_f_ in the shell can be written as the sum of the bending energy *Π*_ben_ and membrane energy *Π*_mem_ thus

1 $$\begin{array}{lll}{{{{{\varPi }}}}}_{\mathrm{f}}&=&{{{{{\varPi }}}}}_{\mathrm{ben}}+{{{{{\varPi }}}}}_{\mathrm{mem}}\\ &=&\frac{1}{2}{\iint }_{{{{\varOmega }}}_{\mathrm{f}}}\left(D{{{{\mathbf{K}}}}}^{\mathrm{T}}\ {\overline{{{{\mathbf{L}}}}}}_{\mathrm{f}}\ {{{\mathbf{K}}}}+J{{{{{\gamma }}}}}^{\mathrm{T}}\ {\overline{{{{\mathbf{L}}}}}}_{\mathrm{f}}\ {{{{\gamma }}}}\right){{{\rm{d}}}}x \, {{{\rm{d}}}}y\\ &=&\frac{D}{2}{\iint }_{{{{\varOmega }}}_{\mathrm{f}}}\left({\kappa }_{x}^{2}+{\kappa }_{y}^{2}+2{\nu }_{\mathrm{f}}{\kappa }_{x}{\kappa }_{y}+\frac{1-{\nu }_{\mathrm{f}}}{2}{\kappa }_{xy}^{2}\right){{{\rm{d}}}}x \, {{{\rm{d}}}}y\\ &&+\frac{{J}_{\mathrm{f}}}{2}{\iint }_{{{{\varOmega }}}_{\mathrm{f}}}\left({\gamma }_{x}^{2}+{\gamma }_{y}^{2}+2{\nu }_{\mathrm{f}}{\gamma }_{x}{\gamma }_{y}+\frac{1-{\nu }_{\mathrm{f}}}{2}{\gamma }_{xy}^{2}\right){{{\rm{d}}}}x \, {{{\rm{d}}}}y,\end{array}$$

where $D={E}_{\mathrm{f}}{h}_{\mathrm{f}}^{3}/[12(1-{\nu }_{\mathrm{f}}^{2})]$ and ${J}_{\mathrm{f}}={E}_{\mathrm{f}}{h}_{\mathrm{f}}/(1-{\nu }_{\mathrm{f}}^{2})$ stand for, respectively, the flexural and extensional rigidities of the shell, and ${\overline{{{{\mathbf{L}}}}}}_{\mathrm{f}}$ represents the dimensionless elastic matrix. The membrane strain tensor and curvature tensor are denoted by *γ* and **K**, respectively. The elastic behaviour of the core can be described by a Winkler-type foundation^25,26^ as

2 $${{{{{\varPi }}}}}_{\mathrm{s}}=\frac{1}{2}{\iint }_{{{{\varOmega }}}_{\mathrm{s}}}{K}_{\mathrm{s}}{w}^{2} \, {{{\rm{d}}}}x \, {{{\rm{d}}}}y,$$

in which ${K}_{\mathrm{s}}={\overline{E}}_{\mathrm{s}}\sqrt{{p}^{2}+{q}^{2}}/2R$ denotes the stiffness of the core^23,27^, *w* stands for deflection, ${\overline{E}}_{\mathrm{s}}={E}_{\mathrm{s}}/(1-{\nu }_{\mathrm{s}}^{2})$, and *p* and *q* represent the wavenumbers along the latitude and longitude directions, respectively.

**Fig. 2:**
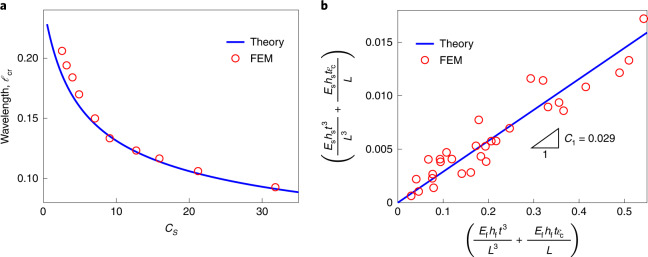
Comparison of theoretical and numerical results. **a**, The critical hexagonal wrinkling wavelength *ℓ*_cr_ as a function of the dimensionless parameter ${C}_{\mathrm{s}}=({E}_{\mathrm{s}}/{E}_{\mathrm{f}}){(R/{h}_{\mathrm{f}})}^{3/2}$ that characterizes the modulus ratio and curvature. **b**, A scaling law (Methods) for the hexagonal-to-chiral mode transition. Our theoretical predictions agree well with FEM simulations, where *C*_1_ denotes the slope. Source data

The critical buckling of a core–shell sphere upon shrinkage is analogous to the hydrostatic instability of a spherical shell where an isotropic stress state remains in the pre-buckling stage, that is, *σ*_*αβ*_*δ*_*αβ*_ = −*σ*, in which *δ*_*αβ*_ is the Kronecker delta, *σ* denotes the external hydrostatic pressure and the Greek indices *α* and *β* take values in {1, 2}. According to Koiter’s theory^24^, elastic stability is primarily determined by the second variation of the total potential energy (*Π*_t_ = *Π*_f_ + *Π*_s_), and one obtains the equilibrium partial differential equations by using the divergence theorem,

3 $$\begin{array}{l}{u}_{,xx}+\frac{1}{2}\left(1-{\nu }_{\mathrm{f}}\right){u}_{,yy}+\frac{1}{2}\left(1+{\nu }_{\mathrm{f}}\right){v}_{,xy}-\frac{1+{\nu }_{\mathrm{f}}}{R}{w}_{,x}=0,\\ {v}_{,yy}+\frac{1}{2}\left(1-{\nu }_{f}\right){v}_{,xx}+\frac{1}{2}\left(1+{\nu }_{\mathrm{f}}\right){u}_{,xy}-\frac{1+{\nu }_{\mathrm{f}}}{R}{w}_{,y}=0,\\ D{\nabla }^{4}w-\frac{{J}_{\mathrm{f}}(1+{\nu }_{\mathrm{f}})}{R}\left({u}_{,x}+{v}_{,y}-2\frac{w}{R}\right)+\sigma {h}_{\mathrm{f}}({w}_{,xx}+{w}_{,yy})\\ \quad\quad\quad\quad +\,{K}_{\mathrm{s}}w=0,\end{array}$$

where a comma in a subscript denotes a partial derivative. As an ansatz, we consider the following forms for the displacements in the critical buckling state:

4 $$\begin{array}{l}u=A\sin (px/R)\cos (qy/R),\\ v=B\cos (px/R)\sin (qy/R),\\ w=C\cos (px/R)\cos (qy/R),\end{array}$$

in which *A*, *B* and *C* refer to the amplitudes of waves. Substituting equations (4) into equations (3) and minimizing with respect to *k* = *p*^2^ + *q*^2^, one obtains the critical conditions for the onset of wrinkling:

5 $$\begin{array}{l}\frac{{h}_{\mathrm{f}}^{2}}{4{c}^{2}{R}^{2}}{k}_{\mathrm{cr}}^{2}-\frac{{\overline{E}}_{\mathrm{s}}R}{4{E}_{\mathrm{f}}{h}_{\mathrm{f}}}\sqrt{{k}_{\mathrm{cr}}}-1=0,\\ \frac{{\sigma }_{\mathrm{cr}}}{{E}_{\mathrm{f}}}=\frac{1}{{k}_{\mathrm{cr}}}+\frac{{h}_{\mathrm{f}}^{2}}{4{c}^{2}{R}^{2}}{k}_{\mathrm{cr}}+\frac{{K}_{\mathrm{s}}{R}^{2}}{{E}_{\mathrm{f}}{h}_{\mathrm{f}}{k}_{\mathrm{cr}}},\\ {\ell }_{\mathrm{cr}}=\frac{2\uppi R}{\sqrt{{k}_{\mathrm{cr}}}},\end{array}$$

where *k*_cr_, *σ*_cr_ and *ℓ*_cr_ denote, respectively, the critical wavenumber, the compressive stress and the wavelength, $c=\sqrt{3(1-{\nu }_{\mathrm{f}}^{2})}$. Here, we define a key dimensionless parameter ${C}_{\mathrm{s}}=({E}_{\mathrm{s}}/{E}_{\mathrm{f}}){(R/{h}_{\mathrm{f}})}^{3/2}$ that characterizes the stiffness ratio of core–shells and the geometric curvature to classify pattern selection. Once the critical wavenumber *k*_cr_ is solved, the theoretical buckling stress and wavelength can be calculated (Fig. 2a). During the natural dehydration process of passion fruit, the moduli of both the surface layer and the soft core may become larger (meaning that the surface layer and the core become stiffer), but we observed that the wrinkling wavelength in experiments (Fig. 1 and Supplementary Video 1) remains almost unchanged, and this critical wavelength *ℓ*_cr_ has some inherent (yet implicit) relation with the modulus ratio *E*_s_/*E*_f_ (equation (5)). Therefore, it is reasonable to approximate in the calculation that the modulus ratio *E*_s_/*E*_f_ remains relatively constant upon dehydration. Note that, although both natural and numerical observations (Fig. 1b,f) show that the buckyball pattern consisting of hexagons and pentagons covers the whole sphere (non-developable surface), the prevailing buckling mode in core–shell spheres is hexagonal. Also within the shallow shell framework (a part of sphere)^24^, it is an analytical challenge to apply both hexagons and pentagons to describe the entire spherical surface. Hence, we assume this dominant hexagonal mode (displacement field) in equation (4), and the critical wrinkling condition based on our theory shows good agreement with numerical simulations. Equation (5), in fact, covers the classical buckling case of a spherical shell without a core (*K*_s_ = 0), for which there are explicit solutions for the critical threshold, that is, *σ*_0_ = *E*_f_*h*_f_/*cR*, *k*_0_ = 2*cR*/*h*_f_ and ${\ell }_{0}=\uppi \sqrt{2R{h}_{\mathrm{f}}/c}$.

Although the critical buckling condition can be predicted analytically by using stability analysis, the secondary bifurcation with the hexagonal-to-chiral mode transition in the post-buckling stage remains a theoretical challenge. Here, we derived a scaling law to provide further insight into such chiral symmetry breaking far beyond the critical threshold (Methods). We assumed that each Y-shaped ridge in the wrinkling hexagons can be regarded as a bilayer system and thus that the chiral ridge instability of core–shell spheres can be simplified as the buckling of bilayered plates under compression. Minimization of the system energy leads to chiral strains that obey the linear relation in Fig. 2b, confirmed by numerical simulations.

### Computation

**Fig. 3:**
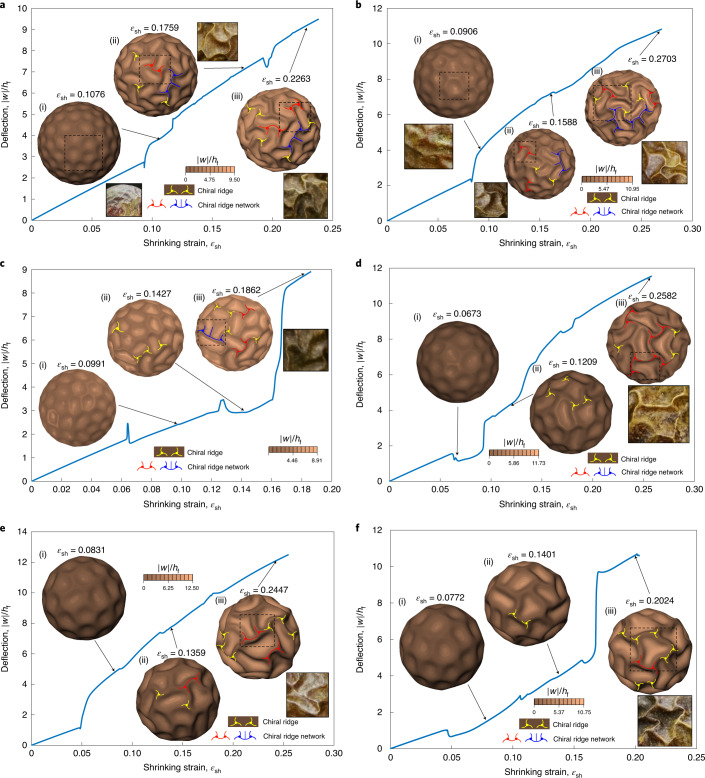
Bifurcation diagrams of post-buckling morphology evolutions in core–shell spheres with different *C*_s_ upon shrinkage. **a**–**f**, Diagrams for *C*_s_ values of 12.7 (**a**), 9.09 (**b**), 7.07 (**c**), 3.98 (**d**), 3.18 (**e**) and 2.55 (**f**), showing the buckyball pattern (with hexagons prevailing) (i) and chiral ridge networks (ii and iii). Excess shrinkage leads to advanced symmetry breaking of the buckyball mode, transforming into the chiral mode and the chiral ridge network eventually. Source data

To trace the whole post-buckling topographic evolution, we applied the finite element method (FEM) by accounting for various geometric and material parameters (Supplementary Section II). The main challenge lies in the solution of nonlinear equations, since multiple solution branches in the post-buckling regime can be connected via multiple bifurcations. Moreover, for instabilities that are extremely localized (for example, the ridge network shown in Fig. 1c,d), there must exist a local transfer of elastic strain energy from one part of the system to the neighbouring regions, and global solution methods may encounter difficulties in convergence. To solve this difficulty, we implemented a pseudodynamic algorithm by introducing velocity-dependent damping and inertial terms, which can be naturally viewed as a perturbation to allow the calculation to pass through the unstable transitions and to trigger chiral symmetry breaking (Methods). The bifurcation portraits of the dimensionless deflection ∣*w*∣/*h*_f_ for various core–shell spheres with different *C*_s_ upon shrinkage are plotted in Fig. 3. Periodic buckyball (with hexagons prevailing) wrinkling patterns with supercritical bifurcation emerge initially at the critical thresholds. Upon further shrinkage, hexagonal-to-chiral mode transitions occur, where Y-shaped ridges in the wrinkling hexagons may buckle into chiral ridges. Neighbouring chiral cellular modes can further interact with each other to form two types of topological network. While symmetry is eventually broken with further shrinkage, leading to universal hexagonal-to-chiral mode transitions, different *C*_s_ values result in different critical thresholds and wavelengths for the buckyball (with hexagon dominating) buckling mode.

### Experiment

**Fig. 4:**
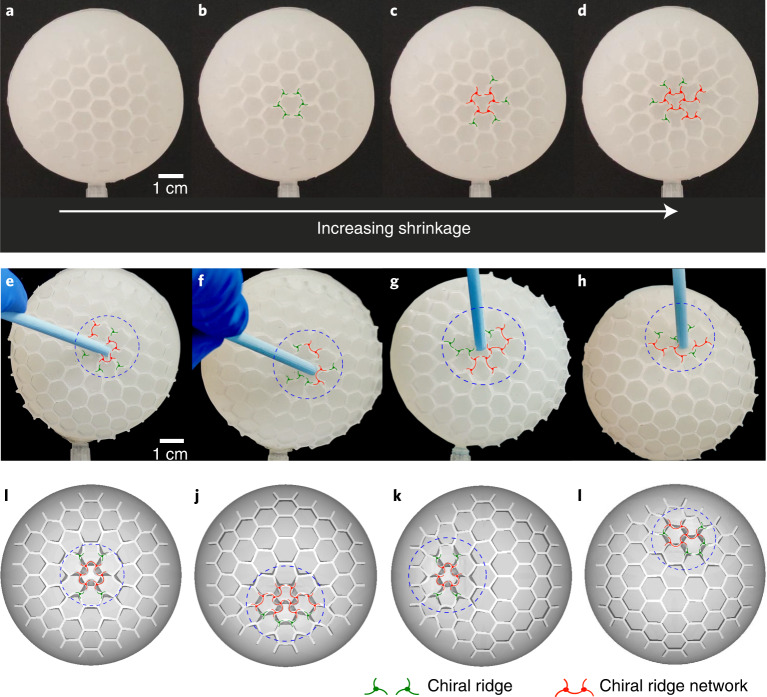
Air extraction-induced chiral topography on curved surfaces. **a**–**d**, The experimental formation of a chiral ridge network with continuous air extraction, showing the hexagonal-to-chiral mode transition with increasing shrinkage of core–shells (Supplementary Video 2). **e**–**l**, The localization of tunable chiral networks on curved surfaces (Supplementary Video 3) triggered by a perturbation (poke by a rod) in experiments (**e**–**h**), consistent with numerical simulations (**i**–**l**).

Guided by this theoretical understanding, we next designed a demonstrative experiment to harness such an instability mechanism to achieve pattern tunability, by using liquid silicone that can solidify into any desired shape in a well-designed mould. We made a spherical shell with a hexagonal pattern on the surface, a cavity and a small hole (diameter ~4 mm) for air extraction to induce shrinkage (Methods). Since silicone has a much lower elastic modulus than passion fruit, the smooth shell structure does not buckle into hexagonal patterns (cannot reach the advanced bifurcation range shown in Fig. 3) but exhibits global deformation upon pressure loading condition by air extraction (Methods and Supplementary Video 5). To focus on the chiral bifurcation and to facilitate instability morphology control at this bifurcation, we fabricated artificial hexagonal patterns on the shell surface. We extracted air slowly (~2 mL s^−1^) from the sample to control the pressure (~10 kPa) so that a state of homogeneous compression could be perfectly achieved. Notably, these well-designed hexagonal networks on the surface of the sample buckle into chiral patterns (Fig. 4a–d and Supplementary Video 2), analogous to the observation of highly dehydrated passion fruits and model predictions (Fig. 1). Furthermore, we can flexibly control the position of local chiral networks by imposing external perturbation as illustrated in Fig. 4e–h (Methods and Supplementary Video 3), consistent with FEM simulations in Fig. 4i–l. These experiments not only demonstrate a hexagonal-to-chiral mode transition, consistent with our theoretical predictions, but also shed light on rational designs of controllable chiral patterns.

### Adaptive grasping

**Fig. 5:**
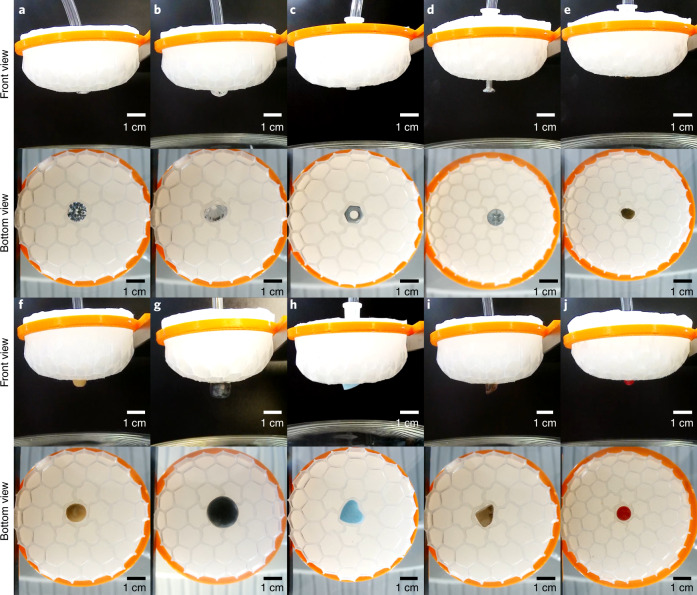
Topographic grasping experiments on objects of different geometry, size and material. **a**–**j**, Grasping of different objects: diamond (**a**,**b**), nut (**c**), screw (**d**), mung bean (**e**), soya bean (**f**), blueberry (**g**), heart-shaped candy (**h**), irregular shaped glass (**i**) and glass ball (**j**). The chiral deformation enables effective, target-adaptive grasping (Supplementary Video 4).

Based on these insights, we show that this perturbation-induced chiral instability can be harnessed to effectively and stably grasp small-sized objects with different geometries and made of different stiff or soft materials. The object to grasp acts as a local perturbation when in contact with the hexagonal-patterned shell and is then adaptively locked by the induced local chiral networks. Similar to the aforementioned experimental setup, we fabricated a hemispherical shell with a hexagonal surface pattern as the main body of the gripper. A small hole was made at the bottom of the cap for air extraction. Then, the whole gripper was fixed onto a lifting frame to steadily control the movement. When the curved hemispherical cap touches the target, the contact perturbation-induced symmetry breaking triggers chiral network localization. The chiral pattern and the interface friction spontaneously adapt to the interactions at the contacting areas, which are naturally influenced by the shape and stiffness of the object, so that different objects can be grasped by this smart locking together with air extraction (Fig. 5, Supplementary Fig. 4 and Video 4). When we restored the pressure difference, that is, inflated the cap cavity, the chiral networks elastically reverted back to hexagons, releasing the grasped object. The contrast experiments showed that the hemispherical caps with a smooth surface (no chiral instability) could not grasp those objects at all (Supplementary Video 5), supporting the critical role of the chiral network localization in the grasping process.

### Discussion

We have unveiled chiral-mode symmetry breaking during excessive shrinkage of core–shell spheres, which can be formulaically described and precisely predicted by our theories and computations, in good agreement with carefully designed experiments. Beyond the critical buckyball wrinkling, chiral ridges emerge on the curved surfaces upon excess deformation, and the neighbouring chiral cellular Y-shaped modes can further interact with each other to form advanced chiral topological networks. The critical buckyball wrinkling conditions can be obtained analytically by using linear stability analysis, while strong nonlinearity (both geometric and material) in the post-buckling regime of shrinking spheres results in considerable difficulties in the theoretical predictions of advanced bifurcations and their associated morphological patterns. Consequently, theoretical analyses on secondary and multiple bifurcations of chiral instability have to resort to dimensional analysis (scaling law) based on certain simplified models. From the computational standpoint, the major challenge in extremely shrinking spheres at large strain is the solution of highly nonlinear equations. The most classical solution method to solve nonlinear static problems is the path-following continuation technique such as that of Riks, while numerical convergence cannot always be ensured for extreme wrinkling problems upon large deformations, since a large number of solution branches can be connected via multiple bifurcations. This fact motivated us to apply the dynamic relaxation method to leap over some localized energy barriers in the nonlinear evolution paths, while the dynamic method cannot straightforwardly predict subcritical bifurcations and hysteresis. Making progress in both theoretical and computational analyses of multiple bifurcations in highly nonlinear evolution paths might require more advanced mathematical approaches.

Inspired by the chiral instability topography induced by local perturbation, we demonstrated an exemplar application of target-adaptive grasping based on chiral localization, while future work may take advantage of smart active materials such as hard-magnetic soft materials and liquid-crystal elastomers to enhance multifunctional designs under multiphysics stimuli. Our results not only provide physical insights into the wrinkling topography of highly deformed core–shell spheres by a universal law but also pave a promising way for realizing multifunctional surfaces by harnessing fruitful topography on curved geometry.

## Methods

### Dimensional analysis of chiral instability

We carried out dimensional analysis to predict the chiral bifurcation of core–shell spheres (Extended Data Fig. 1) upon dehydration (equivalent to thermal shrinkage). Based on the experimental observations and numerical calculations, we assumed that each cellular ridge before chiral instability can be viewed as a layered plate and thus the chiral bifurcation of a cellular ridge can be simplified as the buckling of a bilayer subject to shrinking strain (Extended Data Fig. 1c). Such a plate-like ridge has length *L* and thickness *t* and comprises an upper layer of width *h*_f_ and a lower layer of width *h*_s_. Each layer has a Young’s modulus *E*_*ζ*_, Poisson’s ratio *ν*_*ζ*_ and bending stiffness ${D}_{\zeta }={E}_{\zeta }{t}^{3}/[12(1-{\nu }_{\zeta }^{2})]$, where *ζ* is ‘f’ or ‘s’.

The bending energies of the upper and lower layers can be expressed as

6 $${{{{\mathcal{P}}}}}_{\mathrm{f}}^{\mathrm{b}}=\frac{{D}_{\mathrm{f}}}{2}{\iint }_{{{{\varOmega }}}_{1}}\left[{\left({u}_{\mathrm{f},zz}+{u}_{\mathrm{f},yy}\right)}^{2}+2\left(1-{\nu }_{\mathrm{f}}\right)\left({u}_{\mathrm{f},yz}^{2}-{u}_{\mathrm{f},zz}{u}_{\mathrm{f},yy}\right)\right]{{{\rm{d}}}}{{{\varOmega }}}_{1},$$

7 $${{{{\mathcal{P}}}}}_{\mathrm{s}}^{\mathrm{b}}=\frac{{D}_{\mathrm{s}}}{2}{\iint }_{{{{\varOmega }}}_{2}}\left[{\left({u}_{\mathrm{s},zz}+{u}_{\mathrm{s},yy}\right)}^{2}+2\left(1-{\nu }_{\mathrm{s}}\right)\left({u}_{\mathrm{s},yz}^{2}-{u}_{\mathrm{s},zz}{u}_{\mathrm{s},yy}\right)\right]{{{\rm{d}}}}{{{\varOmega }}}_{2},$$

where *u*_f_ and *u*_s_ denote, respectively, the out-of-plane deflection of the upper and lower layers, while *Ω*_1_ and *Ω*_2_ represent the area of the mid surface of the upper and lower layer, respectively.

As an ansatz, we consider the following forms for the deflections in the chiral buckling state:

8 $${u}_{\mathrm{f}}={{{\varPhi }}}_{\mathrm{f}}\left(z\right)\sin \frac{\uppi }{L}y,$$

9 $${u}_{\mathrm{s}}={{{\varPhi }}}_{\mathrm{s}}\left(z\right)\sin \frac{\uppi }{L}y,$$

where the functions *Φ*_f_(*z*) and *Φ*_s_(*z*) can be expanded into series of exponential decay functions as

10 $${{{\varPhi }}}_{\mathrm{f}}\left(z\right)=\mathop{\sum}\limits_{i}{A}_{\mathrm{f}i}\left({k}_{\mathrm{f}i}z\right),$$

11 $${{{\varPhi }}}_{\mathrm{s}}\left(z\right)=\mathop{\sum}\limits_{i}{A}_{\mathrm{s}i}\left({k}_{\mathrm{s}i}z\right),$$

where *k*_f*i*_ and *k*_s*i*_ are coefficients of the following order:

12 $${k}_{\mathrm{f}i} \sim {k}_{\mathrm{s}i} \sim \frac{1}{L},$$

and the displacement continuity condition is satisfied at the interface of upper and lower layers, that is, *Φ*_f_(*h*_s_) = *Φ*_s_(*h*_s_).

According to equations (8) to (12), one obtains

13 $${u}_{,zz} \sim {u}_{,yy} \sim {u}_{,yz}.$$

Substituting equation (13) into equations (6) and (7), the bending energies read

14 $${{{{\mathcal{P}}}}}_{\mathrm{f}}^{\mathrm{b}} \sim \frac{{E}_{\mathrm{f}}{t}^{3}}{{L}^{4}}{\iint }_{{{{\varOmega }}}_{1}}{\left[\mathop{\sum}\limits_{i}{A}_{\mathrm{f}i}\left({k}_{\mathrm{f}i}z\right)\sin \left(\frac{\pi y}{L}\right)\right]}^{2}{{{\rm{d}}}}y \, {{{\rm{d}}}}z \sim \frac{{E}_{\mathrm{f}}{t}^{3}{h}_{\mathrm{f}}}{{L}^{3}} {a}_{1},$$

15 $${{{{\mathcal{P}}}}}_{\mathrm{s}}^{\mathrm{b}} \sim \frac{{E}_{\mathrm{s}}{t}^{3}}{{L}^{4}}{\iint }_{{{{\varOmega }}}_{2}}{\left[\mathop{\sum}\limits_{i}{A}_{\mathrm{s}i}\left({k}_{\mathrm{s}i}z\right)\sin \left(\frac{\uppi y}{L}\right)\right]}^{2}{{{\rm{d}}}}y \, {{{\rm{d}}}}z \sim \frac{{E}_{\mathrm{s}}{t}^{3}{h}_{\mathrm{s}}}{{L}^{3}} {a}_{2},$$

in which ${a}_{1}=\iint {\left[{\sum }_{i}{A}_{\mathrm{f}i}\left({k}_{\mathrm{f}i}\tilde{z}{h}_{\mathrm{f}}\right)\sin \left(\uppi \tilde{y}\right)\right]}^{2}{{{\rm{d}}}}\tilde{y} \, {{{\rm{d}}}}\tilde{z}$, ${a}_{2}=\iint {\left[{\sum }_{i}{A}_{\mathrm{s}i}\left({k}_{\mathrm{s}i}\tilde{z}{h}_{\mathrm{s}}\right)\sin \left(\uppi \tilde{y}\right)\right]}^{2}{{{\rm{d}}}}\tilde{y} \, {{{\rm{d}}}}\tilde{z}$, $\tilde{y}=y/L$ and $\tilde{z}=z/{h}_{\zeta }$.

The membrane energy can be determined by the in-plane strains given by (note that, for simplicity, the subscript *ζ* has been omitted)

16 $${\varepsilon }_{yy}^{0}=\frac{\partial v}{\partial y}+\frac{1}{2}{\left(\frac{\partial u}{\partial y}\right)}^{2}+{\varepsilon }_{\mathrm{sh}},$$

17 $${\varepsilon }_{zz}^{0}=\frac{\partial w}{\partial z}+\frac{1}{2}{\left(\frac{\partial u}{\partial z}\right)}^{2}+{\varepsilon }_{\mathrm{sh}},$$

18 $${\varepsilon }_{yz}^{0}=0,$$

where *ε*_sh_ is the thermal shrinking strain, and *v* and *w* represent the in-plane displacements in the mid surface along the *y* and *z* directions, respectively, the order of which can be determined by minimizing the membrane energy. Consequently, the in-plane displacements in the mid surface can be approximated as *v* = *By* and *w* = *Cz*, in which *B* and *C* refer to the slopes of variation.

The membrane energies of the upper and lower layers can be expressed as

19 $$\begin{array}{rcl}{{{{\mathcal{P}}}}}_{\mathrm{f}}^{\mathrm{m}}&=&\frac{{E}_{\mathrm{f}}t}{2\left(1-{\nu }_{\mathrm{f}}^{2}\right)}{\iint }_{{{{\varOmega }}}_{1}}\left\{{\left[{\left({\varepsilon }_{yy}^{0}\right)}_{\mathrm{f}}+{\left({\varepsilon }_{zz}^{0}\right)}_{\mathrm{f}}\right]}^{2}+2\left(1-{\nu }_{\mathrm{f}}\right)\right.\\ && \left.\left[{\left({\varepsilon }_{yz}^{0}\right)}_{\mathrm{f}}^{2}+{\left({\varepsilon }_{yy}^{0}\right)}_{\mathrm{f}}{\left({\varepsilon }_{zz}^{0}\right)}_{\mathrm{f}}\right]\right\}{{{\rm{d}}}}{{{\varOmega }}}_{1},\end{array}$$

20 $$\begin{array}{rcl}{{{{\mathcal{P}}}}}_{\mathrm{s}}^{\mathrm{m}}&=&\frac{{E}_{\mathrm{s}}t}{2\left(1-{\nu }_{\mathrm{s}}^{2}\right)}{\iint }_{{{{\varOmega }}}_{2}}\left\{{\left[{\left({\varepsilon }_{yy}^{0}\right)}_{\mathrm{s}}+{\left({\varepsilon }_{zz}^{0}\right)}_{\mathrm{s}}\right]}^{2}+2\left(1-{\nu }_{\mathrm{s}}\right)\right.\\ && \left.\left[{\left({\varepsilon }_{yz}^{0}\right)}_{\mathrm{s}}^{2}+{\left({\varepsilon }_{yy}^{0}\right)}_{\mathrm{s}}{\left({\varepsilon }_{zz}^{0}\right)}_{\mathrm{s}}\right]\right\}{{{\rm{d}}}}{{{\varOmega }}}_{2}.\end{array}$$

According to equations (8) to (12) and (16) to (18), the membrane energies read

21 $${{{{\mathcal{P}}}}}_{\mathrm{f}}^{\mathrm{m}} \sim \frac{{E}_{\mathrm{f}}t{\varepsilon }_{\mathrm{sh}}}{{L}^{2}}{\iint }_{{{{\varOmega }}}_{1}}{\left[\mathop{\sum}\limits_{i}{A}_{\mathrm{f}i}\left({k}_{\mathrm{f}i}z\right)\sin \left(\frac{\uppi y}{L}\right)\right]}^{2}{{{\rm{d}}}}y \, {{{\rm{d}}}}z \sim \frac{{E}_{\mathrm{f}}{h}_{\mathrm{f}}t{\varepsilon }_{\mathrm{sh}}}{L} {a}_{1},$$

22 $${{{{\mathcal{P}}}}}_{\mathrm{s}}^{\mathrm{m}} \sim \frac{{E}_{\mathrm{s}}t{\varepsilon }_{\mathrm{sh}}}{{L}^{2}}{\iint }_{{{{\varOmega }}}_{2}}{\left[\mathop{\sum}\limits_{i}{A}_{\mathrm{s}i}\left({k}_{\mathrm{s}i}z\right)\sin \left(\frac{\uppi y}{L}\right)\right]}^{2}{{{\rm{d}}}}y \, {{{\rm{d}}}}z \sim \frac{{E}_{\mathrm{s}}{h}_{\mathrm{s}}t{\varepsilon }_{\mathrm{sh}}}{L} {a}_{2}.$$

Since the upper and lower layers buckle simultaneously, combining equations (14), (15), (21) and (22) leads to

23 $${{{{\mathcal{P}}}}}_{\mathrm{f}}^{\mathrm{b}}+{{{{\mathcal{P}}}}}_{\mathrm{f}}^{\mathrm{m}} \sim {{{{\mathcal{P}}}}}_{\mathrm{s}}^{\mathrm{b}}+{{{{\mathcal{P}}}}}_{\mathrm{s}}^{\mathrm{m}},$$

namely,

24 $$\left(\frac{{E}_{\mathrm{f}}{h}_{\mathrm{f}}{t}^{3}}{{L}^{3}}+\frac{{E}_{\mathrm{f}}{h}_{\mathrm{f}}t{\varepsilon }_{\mathrm{sh}}}{L}\right) {a}_{1} \sim \left(\frac{{E}_{\mathrm{s}}{h}_{\mathrm{s}}{t}^{3}}{{L}^{3}}+\frac{{E}_{\mathrm{s}}{h}_{\mathrm{s}}t{\varepsilon }_{\mathrm{sh}}}{L}\right) {a}_{2}.$$

Note that *a*_1_/*a*_2_ is a non-negative constant. Based on calculations and equation (24), the scaling law yields the following explicit form for the chiral shrinking strain *ε*_c_:

25 $${C}_{1} \left(\frac{{E}_{\mathrm{f}}{h}_{\mathrm{f}}{t}^{3}}{{L}^{3}}+\frac{{E}_{\mathrm{f}}{h}_{\mathrm{f}}t{\varepsilon }_{\mathrm{c}}}{L}\right)=\left(\frac{{E}_{\mathrm{s}}{h}_{\mathrm{s}}{t}^{3}}{{L}^{3}}+\frac{{E}_{\mathrm{s}}{h}_{\mathrm{s}}t{\varepsilon }_{\mathrm{c}}}{L}\right),$$

where *C*_1_ = 0.029 is a fitting coefficient. The scaling law in equation (25) agrees well with finite element simulations for chiral bifurcation (Fig. 2b).

### Numerical method

We performed finite element simulations in commercial software Abaqus based on parameters similar to experimental observations. Since the deformation of core–shell spheres can be large (up to 30% shrinking strain), we applied the widely used hyperelastic neo-Hookean (nHk) constitutive law for both the surface layer and the soft core, while more sophisticated hyperelastic constitutions such as the Mooney–Rivlin (MR) model were also examined but showed trivial quantitative differences that did not change the substantial nonlinear mechanism of the instability problem. The elastic strain energy density function of the nHk model is defined as

26 $${{{\varPsi }}}_{{{{\rm{nHk}}}}}={C}_{10}\left({I}_{1}-3\right)+\frac{1}{{D}_{1}}{\left(J-1\right)}^{2},$$

in which ${C}_{10}=E/4\left(1+\nu \right)$ and ${D}_{1}=6\left(1-2\nu \right)/E$ are material parameters. The volume change reads $J=\det ({{{\mathbf{F}}}})$, where **F** is the deformation gradient tensor. The first strain invariant reads ${I}_{1}={{{\rm{tr}}}}({{{{\mathbf{F}}}}}^{\mathrm{T}}\cdot {{{\mathbf{F}}}})$. We coupled eight-node hexahedral volume (C3D8R) elements for the soft core and thin shell (S4R) elements for the surface layer by using a ‘tie’ constraint at the interface. Mesh convergence was carefully examined for all simulations. The main challenge is the solution of the nonlinear equations, as numerous post-buckling solution branches can be connected via multiple bifurcations^23,28^. Therefore, we applied the dynamic relaxation method to allow the calculation to pass through the unstable transitions, which introduces velocity-dependent damping (**C**) and artificial inertial (**M**) terms into the static equilibrium equation (**R**(**U**, *λ*) = 0), leading to

27 $${{{\mathbf{M}}}}\frac{{{{{\rm{d}}}}}^{2}{{{\mathbf{U}}}}}{{{{\rm{d}}}}{t}^{2}}+{{{\mathbf{C}}}}\frac{{{{\rm{d}}}}{{{\mathbf{U}}}}}{{{{\rm{d}}}}t}+{{{\mathbf{R}}}}({{{\mathbf{U}}}},\lambda )=0,$$

where **R** is the residual force, **U** denotes unknown variables and *λ* represents an incremental loading parameter. Realistic definitions of mass and damping were not necessary; thus, we set these quantities to obtain optimal convergence of *t* → **U**(*t*) for large values of time *t* (no physical meaning here). When the model is stable (quasi-static), viscous energy dissipation remains quite small such that the artificial damping does not notably perturb the solution. When the system tends to be dynamically unstable, nodal velocities increase, and thus, part of the elastic strain energy released can be dissipated by the damping. A shrinkage load (equivalent to thermal expansion or residual strain) was applied to the core while the surface layer was loading free, which can be expressed as

28 $${\varepsilon }_{\mathrm{sh}}=\alpha {{\Delta }}T{{{\mathbf{I}}}}\quad {{{\rm{with}}}}\quad {{\Delta }}T < 0,$$

where *α*, Δ*T* and **I** stand for the thermal expansion coefficient, temperature change and second-order identity tensor, respectively. The shrinkage load *ε*_sh_ can also be characterized by an isotropic residual strain *ε*_sh_ = *ε*_res_ = −*λ***I**. In the numerical calculations shown in Fig. 1e–h, we took *R*/*h* = 50 and ${C}_{\mathrm{s}}=({E}_{\mathrm{s}}/{E}_{\mathrm{f}}){(R/{h}_{\mathrm{f}})}^{3/2}=9.09$.

### Experimental method for realizing functional chiral surfaces

**Extended Data Fig. 2:**
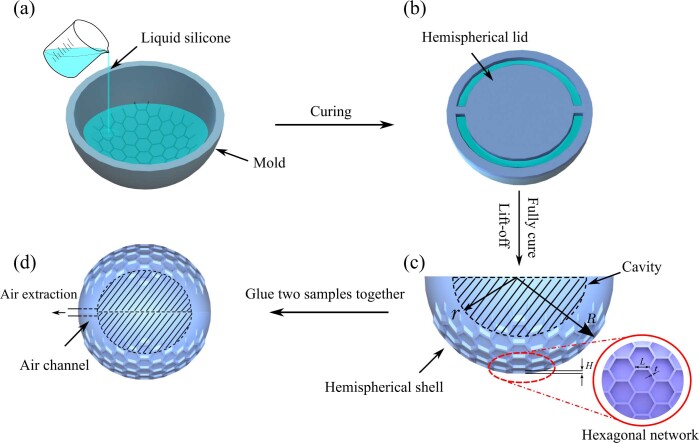
Experimental process of realizing functional chiral topography. (a) Pour liquid silicone on a 3D printed mold with hexagonal network on the surface. (b) Create a cavity in the sample by using a hemispherical cover when the liquid silicone is curing. (c) A silicon hemispherical shell with hexagonal pattern on the surface. (d) Two hemispherical shells with hexagonal network are glued and can be separated, with a channel connecting the internal cavity and external tube for air extraction.

To realize flexible tunability of chiral patterns and to further harness the hexagonal-to-chiral mode transition for achieving smart surfaces, we designed demonstrative experiments based on air extraction from silicon core–shell spheres. The simple experimental system consists of two combined hemispherical caps with a channel connecting the internal cavity and an external tube for air extraction. To achieve a hexagonal network on the surface of the hemispherical cap, we designed a mould with a hexagonal network by applying three-dimensional printing technology. Then, we poured in two-part liquid silicone (Hongyejie Technology Co. Ltd.) in 1:1 mass ratio. Liquid silicone needs to stand for 3 hours at 25 °C to cure fully. To create a cavity in the centre of the sample, we applied a hemispherical lid with a diameter slightly smaller than the outer diameter to cover the bottom of the mould when the liquid silicone was curing. After the liquid silicone had cured and was demoulded, we glued two identical hemispherical caps together. The typical parameters of the samples were an outer diameter of 2*R* = 70 mm, a diameter of the inner cavity of 2*r* = 58 mm and a hexagonal cellular length of *L* = 4.33 mm, height of *H* = 2.61 mm and thickness of *t* = 0.75 mm. The experimental procedure to realize functional chiral surfaces is illustrated in Extended Data Fig. 2. The inner cavity of the samples was pumped out and depressurized to create a state of homogeneous shrinkage. To demonstrate the effects of shrinkage on the hexagonal-to-chiral mode transition, we slowly exhausted the air in the samples to mimic dehydration-induced shrinkage of passion fruit. When the samples deformed elastically to certain values, the hexagonal network lost stability and buckled into a chiral topography (Fig. 4a–d). Note that this mode transition is reversible when the air re-enters the sample and the pressure difference is restored. To further illustrate the tunability of the chiral localization, we applied a small disturbance (poke by a rod) somewhere on the surface to trigger the hexagonal-to-chiral mode transformation (Fig. 4e–h) while the sample was subjected to homogeneous shrinkage, which was in good agreement with finite element simulations (Fig. 4i–l). This strategy can provide enlightenment for the design of programmable functional surfaces such as adaptive grasping based on chiral localization.

### Chiral topography for adaptive grasping

Based on the aforementioned experiment, we present a target-adaptive gripper which can grasp small objects based on a hexagonal-to-chiral mode transformation. Simple structure, easy control, shape adaptation and filterable grasping are prominent advantages of the chiral gripper. The gripper system consists of a hemispherical shell with hexagonal topography, an air channel and a lifting frame that can move up and down (Supplementary Fig. 3). The air channel and the hemispherical part constitute a cavity structure, the former being connected to an external exhaust device to trigger the hexagonal-to-chiral mode transition by air extraction. The lifting frame is combined with the cap to control the motion. The working principle of the gripper is introduced as follows: The lifting frame descends to make the gripper approach a target. When the hexagonal network on the curved surface touches the object, the contact perturbation triggers the hexagonal-to-chiral topographic deformation that can well fit with the targeted shape. Then, the exhaust device begins to pump air. With increasing air extraction, the chiral topography can lock the object tightly to achieve a stable grasp. Finally, the object leaves the desk when raising the lifting frame. When the pressure difference is restored, the chiral topography elastically reverts back to hexagonal networks, releasing the grasped object. We carried out topographic grasping experiments on stiff or soft objects of different shapes and sizes (Fig. 5 and Supplementary Fig. 4). Our experiments showed that the gripper can smartly and stably grasp various small-sized objects. To further demonstrate the crucial role played by the chiral topography in robust grasping, we performed contrast experiments by making a hemispherical cap with a smooth surface. Except for the lack of the initial hexagonal network on the surface, the other parameters of the gripper remained exactly the same as in the aforementioned grasping experiments. With the smooth surface, the targets slid off, leading to failure of effective grasping (Supplementary Video 5). Our experiments not only prove the critical role of the chiral topography in effective, target-adaptive grasping but also shed light on smart gripper designs.

### Reporting summary

Further information on research design is available in the Nature Research Reporting Summary linked to this article.

### Supplementary information

Supplementary Information Supplementary Figs. 1–4 and Table 1.

Reporting summary

Peer review file

Supplementary Video 1 Natural dehydration of passion fruit and numerical simulation.

Supplementary Video 2 Hexagonal-to-chiral topography transition induced by air extraction.

Supplementary Video 3 Chiral topography formation induced by surface disturbance.

Supplementary Video 4 Chiral topography for adaptive grasping.

Supplementary Video 5 Contrast experiments with smooth surface

### Source data

Source Data Fig. 2 FEM source data

Source Data Fig. 3 FEM source data

## Data Availability

Source data for the FEM computations shown in Figs. 2 and 3 are available with this manuscript.

## Code availability

The code used in this study can be obtained from Zenodo^29^.

## References

[1] Yin J, Cao Z, Li C, Sheinman I, Chen X (2008). Stress-driven buckling patterns in spheroidal core/shell structures. Proc. Natl Acad. Sci. USA.

[2] Yin J, Chen X, Sheinman I (2009). Anisotropic buckling patterns in spheroidal film/substrate systems and their implications in some natural and biological systems. J. Mech. Phys. Solids.

[3] Li B, Jia F, Cao YP, Feng XQ, Gao H (2011). Surface wrinkling patterns on a core–shell soft sphere. Phys. Rev. Lett..

[4] Dervaux J, Ben Amar M (2008). Morphogenesis of growing soft tissues. Phys. Rev. Lett..

[5] Huang C, Wang Z, Quinn D, Suresh S, Hsia KJ (2018). Differential growth and shape formation in plant organs. Proc. Natl Acad. Sci. USA.

[6] Xu F, Fu C, Yang Y (2020). Water affects morphogenesis of growing aquatic plant leaves. Phys. Rev. Lett..

[7] Ben Amar M, Jia F (2013). Anisotropic growth shapes intestinal tissues during embryogenesis. Proc. Natl Acad. Sci. USA.

[8] Ciarletta P, Balbi V, Kuhl E (2014). Pattern selection in growing tubular tissues. Phys. Rev. Lett..

[9] Ciarletta P (2013). Buckling instability in growing tumor spheroids. Phys. Rev. Lett..

[10] Tallinen T (2016). On the growth and form of cortical convolutions. Nat. Phys..

[11] Brau F (2011). Multiple-length-scale elastic instability mimics parametric resonance of nonlinear oscillators. Nat. Phys..

[12] Stoop N, Lagrange R, Terwagne D, Reis PM, Dunkel J (2015). Curvature-induced symmetry breaking determines elastic surface patterns. Nat. Mater..

[13] Katifori E, Alben S, Cerda E, Nelson DR, Dumais J (2010). Foldable structures and the natural design of pollen grains. Proc. Natl Acad. Sci. USA.

[14] Holland M, Budday S, Goriely A, Kuhl E (2018). Symmetry breaking in wrinkling patterns: Gyri are universally thicker than sulci. Phys. Rev. Lett..

[15] Bowden N, Brittain S, Evans AG, Hutchinson JW, Whitesides GM (1998). Spontaneous formation of ordered structures in thin films of metals supported on an elastomeric polymer. Nature.

[16] Rogers JA, Someya T, Huang Y (2010). Materials and mechanics for stretchable electronics. Science.

[17] Pocivavsek L (2018). Topography-driven surface renewal. Nat. Phys..

[18] Pikul JH (2017). Stretchable surfaces with programmable 3D texture morphing for synthetic camouflaging skins. Science.

[19] Siéfert E, Reyssat E, Bico J, Roman B (2019). Bio-inspired pneumatic shape-morphing elastomers. Nat. Mater..

[20] Terwagne D, Brojan M, Reis PM (2014). Smart morphable surfaces for aerodynamic drag control. Adv. Mater..

[21] Cao G, Chen X, Li C, Ji A, Cao Z (2008). Self-assembled triangular and labyrinth buckling patterns of thin films on spherical substrates. Phys. Rev. Lett..

[22] Breid D, Crosby AJ (2013). Curvature-controlled wrinkle morphologies. Soft Matter.

[23] Xu F, Zhao S, Lu C, Potier-Ferry M (2020). Pattern selection in core–shell spheres. J. Mech. Phys. Solids.

[24] van der Heijden, A. M. A. *W.T. Koiter’s Elastic Stability of Solids and Structures* (Cambridge Univ. Press, 2009).

[25] Biot MA (1937). Bending of an infinite beam on an elastic foundation. J. Appl. Mech..

[26] Allen, H. G. *Analysis and Design of Structural Sandwich**Panels* (Pergamon, 1969).

[27] Zhao Y, Cao Y, Feng XQ, Ma K (2014). Axial compression-induced wrinkles on a core-shell soft cylinder: Theoretical analysis, simulations and experiments. J. Mech. Phys. Solids.

[28] Groh RMJ, Avitabile D, Pirrera A (2018). Generalised path-following for well-behaved nonlinear structures. Comput. Methods Appl. Mech. Eng..

[29] Xu, F., Huang, Y., Zhao, S. & Feng, X. Q. Chiral topographic instability in shrinking spheres. *Zenodo*10.5281/zenodo.7025830 (2022). 10.1038/s43588-022-00332-y PMC10766550 38177274

