# Supplementary material for: Chiral topographic instability in shrinking spheres
Source: Nat Comput Sci. 2022 Oct 24;2(10):632–40. doi: 10.1038/s43588-022-00332-y (PMC10766550; doi:10.1038/s43588-022-00332-y)
Supplement: Supplementary file 1 — Supplementary Figs. 1–4 and Table 1. [file 43588_2022_332_MOESM1_ESM.pdf]

nature computational science

Article https://doi.org/10.1038/s43588-022-00332-y

Chiral topographic instability in shrinking spheres

In the format provided by the authors and unedited

Here we provide more details about discussions on postbuckling morphology evolutions with further experiments of natural dehydration and computational results, and experimental designs for realizing functional chiral surfaces and topographic grasping.

I. NATURAL DEHYDRATION EVOLUTIONS OF PASSION FRUITS

We looked into morphology evolutions of passion fruits upon natural dehydration (see Supplementary Fig. 1). During shrinking process, the initial smooth surface buckles into periodic hexagons (prevailing mode) and then a hexagonal-to-chiral ridge mode transition appears with continuous dehydration. Furthermore, when the samples are excessively dehydrated, the neighboring chiral cellular ridge mode can further interact with each other to form topological ridge networks. Despite there may exist material or geometric defects in passion fruits as well as environmental influence in the natural dehydration process, analogous topographic evolutions, compared to our theoretical predictions, can be observed.

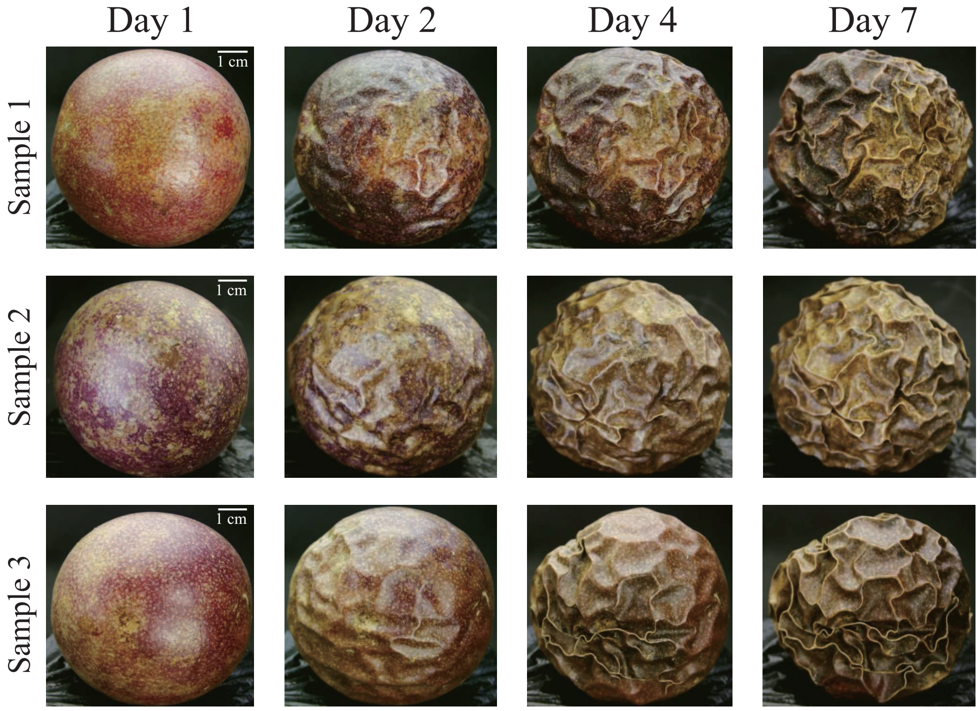

Supplementary Figure 1. Surface morphology evolutions of passion fruits (three samples) upon natural dehydration process. Observations suggest that the spherical core-shell fruits experience a smooth-hexagonal-chiral mode transition (see Supplementary Video 1).

II. COMPUTATIONAL POSTBUCKLING MORPHOLOGY EVOLUTIONS OF SHRINKING CORE-SHELL SPHERES

We computed postbuckling morphology evolutions of diverse core-shell spheres with different geometric and material parameters that are listed in Supplementary Table 1. A key factor to determine pattern selection can be characterized by a dimensionless parameter $C_s = (E_s/E_f)(R/h_f)^{3/2}$, in function of radius/thickness ratio R/h_f and modulus ratio E_s/E_f. Hence, different cases with representative geometric and material properties were considered for computations, some of which have a relatively big C_s while the others hold a small value.

Supplementary Table 1. Geometric and material properties of various core-shell spheres.

Core-shell	E_f/E_s	ν_f/ν_s	R/h_f	C_s	ℓ_{cr}/ℓ_0
I	50/1.8	0.3/0.48	0.5/0.01	12.7	0.122/0.244
II	70/1.8	0.3/0.48	0.5/0.01	9.09	0.135/0.244
III	90/1.8	0.3/0.48	0.5/0.01	7.07	0.145/0.244
IV	160/1.8	0.3/0.48	0.5/0.01	3.98	0.169/0.244
V	200/1.8	0.3/0.48	0.5/0.01	3.18	0.178/0.244
VI	250/1.8	0.3/0.48	0.5/0.01	2.55	0.186/0.244
VII	500/1.8	0.3/0.48	0.5/0.01	1.27	0.209/0.244

Computational postbuckling morphology evolutions of diverse core-shell spheres are plotted in Fig. 3 in the main text. Different C_s results in different critical thresholds and wavelengths for buckyball (hexagons dominating) buckling mode, while the symmetry of patterns is eventually broken with the increase of shrinkage, resulting in general hexagonal-to-chiral mode transitions, except a relatively soft core-shell sphere with $C_s = 1.27$ where the deformed shapes are isolated circular dimples without significant interaction with each other (see Supplementary Fig. 2). Such localization mode was widely observed in spherical shell (without a core) buckling [1, 2]. This bifurcation scenario is found general here since all tested core-shell systems with $C_s < 1.27$ follow the similar bifurcation diagram with localized dimples.

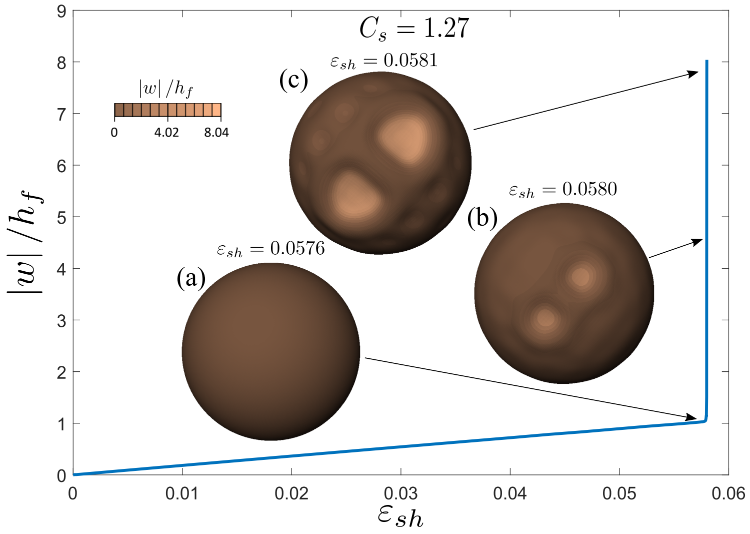

Supplementary Figure 2. Bifurcation curve of postbuckling morphology evolution of core-shell VII with $C_s = 1.27$ upon shrinkage. The deformed shapes are isolated dimples without networking.

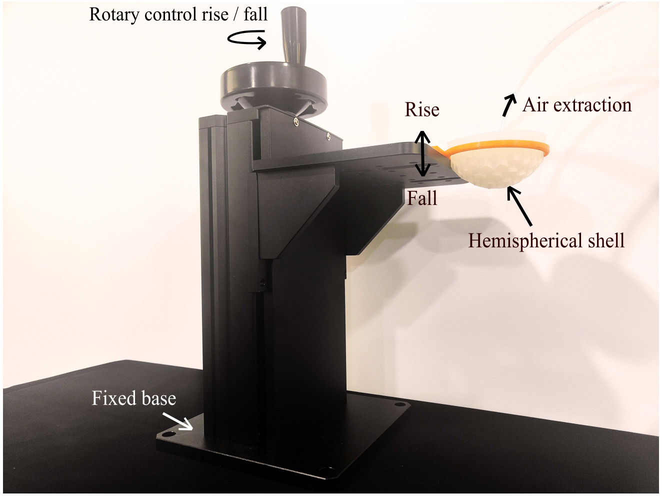

Supplementary Figure 3. The gripper system consists of of a hemispherical shell with hexagonal topography, an air channel and a lifting frame.

---

[1] B. Audoly and J.W. Hutchinson, Localization in spherical shell buckling, J. Mech. Phys. Solids **136**, 103720 (2020).

[2] F. Xu, S. Zhao, C. Lu, and M. Potier-Ferry, Pattern selection in core-shell spheres, J. Mech. Phys. Solids **137**, 103892 (2020).

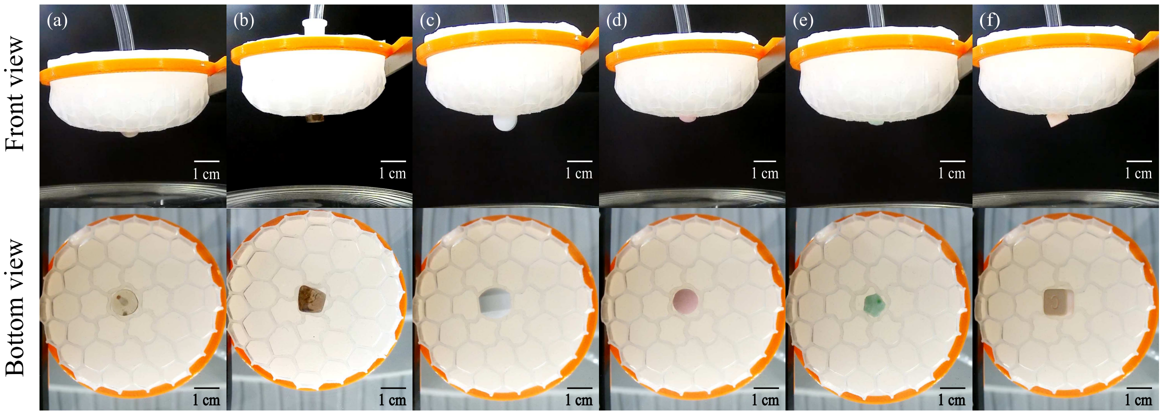

Supplementary Figure 4. Topographic grasping experiments on objects of different geometries, sizes and materials: (a) glass ball, (b) rock, (c) cylindrical candy, (d) cake-shaped candy, (e) pentagon-shaped candy (f) cube-shaped candy. The deformation of chiral network enables the effective, target-adaptive grasping (see Supplementary Video 4).
